# Effect of Intermittent Fasting on Glycaemic Control in Patients With Type 2 Diabetes Mellitus: A Systematic Review and Meta-analysis of Randomized Controlled Trials

**DOI:** 10.17925/EE.2023.19.1.25

**Published:** 2023-01-17

**Authors:** Suresh K Sharma, Shiv Kumar Mudgal, Sanjay Kalra, Rakhi Gaur, Kalpana Thakur, Rajat Agarwal

**Affiliations:** 1. College of Nursing, All India Institute of Medical Sciences, Jodhpur, Rajasthan, India; 2. College of Nursing, All India Institute of Medical Sciences, Deoghar, Jharkhand, India; 3. Department of Endocrinology, Bharti Hospital and BRIDE, Karnal, Haryana, India; 4. College of Nursing, All India Institute of Medical Sciences, Rishikesh, Uttarakhand, India; 5. Department of Cardiothoracic Surgery, All India Institute of Medical Sciences, Deoghar, Jharkhand, India

**Keywords:** Diabetes mellitus, diet habits, glycated hemoglobin, glycemic control, intermittent fasting, meta-analysis

## Abstract

**Background:** Type 2 diabetes mellitus (T2DM) is a severe public health issue notably impacting human life and health expenditure. It has been observed in literature that intermittent fasting (IF) addresses diabetes and its underlying cause, which benefits people with diabetes. Therefore, this study aimed to evaluate the effectiveness of IF treatment on glycaemic control in people with T2DM compared with control group. **Methods:** Systematic review and meta-analysis of interventional studies among patients with T2DM with glycated haemoglobin (HbA1c) as an outcome was performed. A comprehensive search of electronic databases, including PubMed, Embase and Google Scholar, for articles published before 24 April 2022, was done. Studies reporting 24 hours of complete fasting or intermittent restricted energy intake (feeding permitted for only 4–8 hours daily, with 16–20 hours of fasting) and reporting changes in HbA1c and fasting glucose levels were eligible. Meta-analysis was performed using Cochrane’s Q statistic and the I^2^ statistical approach. **Results:** Eleven studies (13 arms) measuring the effect of IF on patients’ HbA1c level were analysed. There was no statistically significant difference between IF and control groups (Standardized mean difference [SMD] -0.08, 95% confidence interval [CI] -0.20 to 0.04;p=0.19, I^2^=22%). Overall, seven studies on patients’ fasting blood glucose were analysed, and the meta-analysis revealed no significant difference between the two groups i.e. IF and control groups (SMD 0.06, 95% CI -0.25 to 0.38;p=0.69, I^2^=76%). **Conclusion:** IF and usual diet pattern have no difference in terms of glycaemic control. Although, IF may be used as a preventative diet pattern in the pre-diabetic population, as it works well in the long-term to achieve controlled sugar levels. Study registration: The protocol of this study was registered in The International Prospective Register of Systematic Reviews (PROSPERO) with a registration number CRD42022328528.

Type 2 diabetes mellitus (T2DM) is a severe public health issue notably impacting human life and health expenditure. Around 9.3% (463 million people) of the global population were living with diabetes in 2019, and this is projected to increase to 10.2% (578 million people) by 2030 and 10.9% (700 million people) by 2045.^1,2^ Diabetes impacts functional capacity and quality of life, and ultimately causes significant morbidity and premature mortality. In 2019, diabetes was the tenth biggest cause of death worldwide, directly causing an estimated 1.5 million deaths.^3^ Despite lifestyle treatments such as a healthy diet, frequent physical activity and maintaining a normal body weight being critical pillars of diabetes management, achieving persistent glycaemic control with non-pharmacological techniques is difficult.^4,5^

Recent studies have investigated the benefits of intermittent fasting (IF), which involves repeatedly and purposefully interrupting or drastically reducing energy intake for a period, for people with obesity and T2DM. IF has also been suggested as a glycaemic control and weight loss strategy with additional cardio-metabolic benefits.^6–9^Although it has not yet been standardized, intermittent or short-term energy restriction through very low-calorie diets is a common IF regimen.^4,5^ Time-restricted feeding, which allows for only 4–8 hours of feeding per day (16–20 hours of fasting per day), is one of the most popular IF regimens.^10,11^Other popular IF techniques include alternate-day fasting and periodic fasting, which call for a circular diet that includes fasting for 1 or 2 days per week (burning ≤25% of the required calories) and eating normally for the rest of the week.^12–14^

The impact of IF has been observed on a range of health outcomes including risk factors for metabolic diseases, such as weight, blood pressure, waist circumference, body fat, lipid distribution and blood glucose.^15–18^ Previous studies on people with T2DM have shown that IF can result in comparable weight loss and glycated haemoglobin (HbA1c) reduction as traditional dietary recommendations.^19–22^ However, in some randomized crossover experiments, IF had no impact on lipid and glucose metabolism.^23–24^

These findings demonstrate that IF inconsistently impacts numerous metabolic parameters. Furthermore, the small sample sizes of these studies prevent drawing of firm conclusions. Therefore, a thorough and methodical meta-analysis that includes all eligible randomized controlled trials, a large sample size, and a range of IF types is needed to ascertain the effectiveness of IF interventions on glycaemic control in people with T2DM. This comprehensive review and meta-analysis evaluates the effect of IF treatments on glycaemic control in people with T2DM.

## Materials and methods

This systematic review was submitted to the International Prospective Register of Systematic Reviews and followed the Preferred Reporting Items for Systematic Reviews and Meta-Analysis (PRISMA) guidelines.^25^ The protocol of this study was registered (PROSPERO ID: CRD42022328528).

### Databases and search strategy

This meta-analysis was done and presented in accordance with the PRISMA standards. We conducted a comprehensive search of electronic databases, including PubMed, Embase and Google Scholar, for articles published before 24 April 2022, regardless of area. In addition, the reference lists of particular articles were examined. As search terms, we used intermittent fasting, intermittent energy restriction, type 2 diabetes, HbA1c and fasting blood glucose.

### Inclusion and exclusion criteria

We selected articles that met the following standards: (1) the participants had T2DM and were at least 18 years old; (2) interventional research, which may consist of randomized parallel-arm or crossover trials; (3) intervention: i) 24 hours of complete fasting; ii) intermittent restricted energy intake; iii) time-restricted feeding (feeding permitted for only 4–8 hours daily with 16–20 hours of fasting); (4) the IF intervention could be applied on alternate days, twice weekly or continuously and was compared with standard dietary recommendations consisting of regular eating hours (control group); (5) the changes in HbA1c were recorded; and (6) the duration of the trial exceeded 6 weeks.

The criteria for exclusion were: (1) trials without a control group, or other study designs; (2) studies that lack a HbA1c factor as an outcome or did not provide enough information; (3) non-human samples, reviews and case studies; (4) studies that were reported in a language other than English; (5) absence of time restrictions for intermittent energy restrictions (IER) and fasting. Cienfuegas et al. and Harvie et al. have performed studies with outcomes measured at different levels or time. Both of their outcomes were included in our meta-analysis and stated as Cienfuegas et al. [a] & [b]; Harvie et al. [a] & [b]. Therefore, the total included studies in our metaanalysis are 11 but HbA1c level outcomes shows analysis of 13 studies.

### Data extraction

Two researchers separately reviewed databases and deleted redundant studies. Pairs of independent reviewers first looked over the titles and abstracts of all articles that met the inclusion criteria, before reading the entire text of applicant studies. Disputes concerning a study’s inclusion were discussed and resolved by a third reviewer. The reference lists of the chosen papers were also examined. Independent data extraction was done by two researchers. For each included study, the following parameters were extracted: basic information (first author, year, title and country), clinical features (participants’ characteristics, dietary habits, intervention follow-up duration and results), and method and design (randomization procedure and data analysis technique). The variation in HbA1c and fasting glucose levels were the most crucial finding. We emailed the corresponding author when we needed information specific to the study.

### Risk of bias assessment

Using the updated Cochrane risk of bias assessment tool for randomized trials,^26^ two independent reviewers assessed the likelihood of bias in trials based on the outcomes (HbA1c or fasting glucose). The Cochrane Handbook for Systematic Reviews categorizes the risk of bias for each domain as low, high or unclear based on the signal questions for each item.

### Data analysis

The mean difference between before and after IF implementation and their respective 95% confidence intervals (CIs) were used to evaluate the effects of IF on HbA1c. To measure trial heterogeneity, Cochrane’s Q statistic and the I^2^ statistical approach were applied. A random-effect meta-analysis model was used if the pertinent p value was less than 0.05 and I^2^ was higher than 50%. Otherwise, a fixed-effect model was chosen. For each outcome, funnel plots depicting effect sizes versus standard errors were constructed and visually evaluated to assess the probability of bias. For statistical analysis, we used RevMan 5.4 software (Cochrane, London, UK).

## Results

### Study characteristics

**Figure 1.**
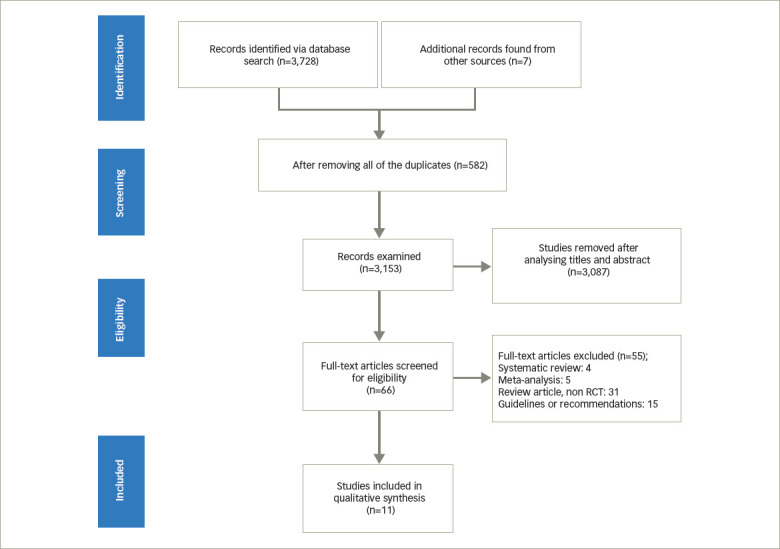
Study selection, assessment and inclusion

*Figure 1* depicts a flowchart of the literature search procedure. Using this search method, we evaluated 3,153 studies after deleting 582 duplicates. Screening titles and abstracts eliminated an additional 3,087 articles. The remaining 66 citations’ entire texts were evaluated in greater detail to determine their eligibility. A further 31 articles were excluded because they were review articles, 15 studies were excluded because they were guidelines or recommendations, and nine studies were excluded because they were systematic reviews and metaanalyses. Eleven studies including 879 patients were included in the final data synthesis.^22,27–36^

*Table 1* presents the features of and interventions used in the 11 selected studies. All were randomized parallel-arm trials, with the exception of one crossover trial and one non-inferiority trial. The studies were published between 1991 and 2020. The sample sizes in these trials were 33–137, and the duration of the interventions was 10–12 months. The mean age of the patients was 45.2–65.5 years, and 38.77% were male. The IF interventions used varied, with one study evaluating time-restricted feeding, two studies evaluating caloric restriction, four studies evaluating intermittent energy restriction and four studies evaluating extremely low-caloric diet.

### Risk of bias and quality assessment of studies

**Figure 2.**
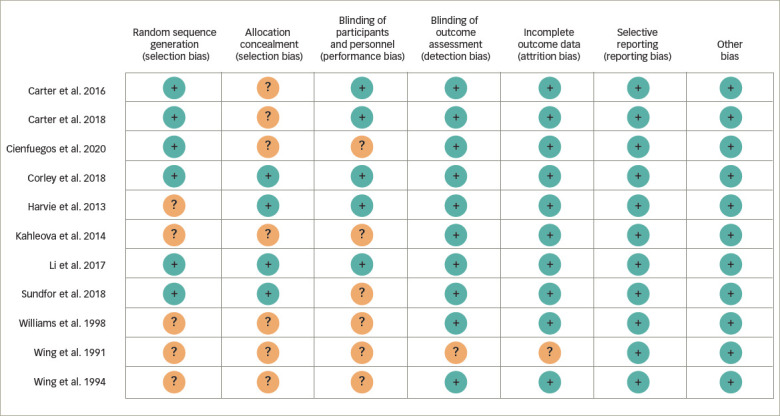
Risk of bias assessment of the included studies using the Cochrane risk of bias tool for randomized trials on glycaemic control outcomes across seven domains

*Figure 2* and the supplementary table provide information on all Cochrane risk-assessment domains and methodology findings. The majority of studies had a minimal risk of bias. In case of any missing information from the study findings and after receiving responses from the corresponding authors of included studies, all authors of this analysis reached a consensus on the next steps.

**Table 1.** Characteristics of studies included in the meta-analysis

Study	Country	Design	Participants, n (l:C)	Mean± SDage in years I (C)	Sex, female:male	Study duration, weeks	intervention	Control	Outcomes
Cienfuegos et al. 2020 [a]^27^	USA	Randomized controlled	38 (19:19)	49±2 (45±2)	34:4	10	4-hourTRF: eating only 3-7 pm (without having to count calories)	Usual diet pattern with no meal timing restrictions	FM, SBP, DBP, TG, LDL-C, HDL-C, FBG, Fins, HbAlc, HOMA-IR
Cienfuegos et al. 2020 [b]^27^	USA	Randomized controlled	39 (20:19)	46±3 (45±2)	36:3	10	6-hourTRF: eating only 1-7 pm (without having to count calories)	Usual diet pattern with no meal timing restrictions	FM, SBP, DBP, TG, LDL-C, HDL-C, FBG, Fins, HbAlc, HOMA-IR
Carter et al. 2018^28^	Australia	Randomized noninferiority	137 (70:67)	61±9 (61 ±9)	77:60	52	IER: 500-600 kcal/day, followed for two non-consecutive days/week (usual diet for the other 5 days)	CER	Weight, FM, BMI, HbAlc
Corley et al. 2018^30^	New Zealand	Randomized controlled	37 (19:18)	58 (62)	15:22	12	Non-consecutive days caloric restriction: 5:2 schedule a VLCD for 2 days/week	CER	Weight, WC, FM, BMI, SBP, DBRTC, TG, LDL-C, HDL-C, FBG, HbAlc
Sundfor et al. 2018^34^	Norway	Randomized controlled	112 (54:58)	49.9±10.1 (47.5±11.6)	56:56	26	IER: 400/600 kcal/day(female/male) on each of two non-consecutive days/week and consume as usual diet for next 5 days/week	CER	Weight, WC, BMI, SBP, DBP, TC, TG, LDL-C, HDL-C, FBG, HbAlc
Li etal. 2017^33^	Germany	Randomized controlled pilot	46 (23:23)	64.7±7.0 (65.5±5.7)	N/A	16	2 pre-fasting days with moderate caloric restriction followed by 7 modified fasting days and subsequent stepwise re-introduction of ordinary food items over 3 days	Mediterranean diet	Weight, WC, BMI, SBP, DBP, TC, TG, LDL-C, HDL-C, FBG, Fins, HbAlc, HOMA-IR
Carter et al. 2016^29^	Australia	Parallel, randomized, controlled	63 (31:32)	61 ±8 (62±9)	33:30	12	IER: 1,670-2,500 kJ/day for 2 days each week, and the remaining 5 days included habitual eating	CER	Weight, FM, HbAlc
Harvie et al. 2013 [a]^31^	USA	Single-centre randomized	77 (37:40)	45.6±8.3 (47.9±7.7)	37:40	12	IECR: restrict energy and carbohydrates on 2 consecutive days each week and Mediterranean-type diet for the remaining 5 days of the week	Daily energy restriction	Weight, WC, SBP, TC, LDL-C, HDL-C, FBG, Fins, HbAlc, HOMA-IR
Harvie et al. 2013 [b]^31^	USA	Single-centre randomized	78 (38:40)	48.6±7.3 (47.9±7.7)	38:40	12	IECR and *ad libitum* protein and fat	Daily energy restriction	Weight, WC, SBP, TC, LDL-C, HDL-C, FBG, Fins, HbAlc, HOMA-IR
Kahleova et al. 2014^32^	Czech Republic	Randomized crossover	54 (27:27)	59.4±7.0	25:29	12	12 weeks of two meals/day (breakfast 6-10 am and lunch 12-4 pm)	12 weeks of six meals/day (breakfast, lunch, dinner ± 3 snacks)	Weight, WC,TC, HDL-C, LDL-C, FBG, HbAlc
Williams et al. 1998 [a]^35^	USA	Randomized parallel-arm	36 (18:18)	50.3±8.6 (54.1 ±7.0)	11:7	20	VLCD (400-600 kcal/day) 5 consecutive days on Week 2, then VLCD 1 day/week and regular diet (1,500-1,800 kcal) 6 days/week for next 15 weeks	CER: 1,500-1,800 kcal/day every day	Weight,TC,TG, LDL-C, HDL-C, HbAlc, Fins
Williams et al. 1998 [b]^35^	USA	Randomized parallel-arm	36 (18:18)	51.4±7.9 (54.1 ±7.0)	9:9	20	IER (5 days/week): 400-600 kcal/day on fast day every 5 weeks and 1,500-1,800 kcal/day on feed days	CER: 1,500-1,800 kcal/day every day	weight,TC,TG, LDL-C, HDL-C, HbAlc, Fins
Wing et al. 1994^22^	USA	Randomized parallel-arm	93 (45:48)	51.8±9.7	60:33	50	VLCD (400-500 kcal/day) in Weeks 0-12 and at 24, and LCD (1,000-1,200 kcal/day) for remaining weeks	LCD (1,000-1,200 kcal/day) throughout	Weight, TC,TG, LDL-C, HDL-C, SBP, DBP, HbAlc, FBG
Wing et al. 1991^36^	USA	Randomized parallel-arm	33 (17:16)	51.2±9.7	8:25	20	VLCD (400 kcal/day) in Weeks 5-12 (run-in period 0-5), behaviour therapy ±1,000-1,500 kcal/day for remaining weeks	Behaviour therapy ± 1,000-1,500 kcal/day for 20 weeks	Weight,TC,TG, LDL-C, HDL-C, HbAlc, FBG

*BMI = body mass index; C = control; CER = continuous energy restriction; DBF = diastolic blood pressure; FBG = fasting blood glucose; Fins = fasting insulin; FM = fat mass; HbAlc = glycated haemoglobin; HDL-C = high-density lipoprotein cholesterol; HOMA-IR = Homeostatic Model Assessment for Insulin Resistance; I = intervention; IECR = intermittent energy and carbohydrate restriction; IER = intermittent energy restriction; LCD = low-calorie diet; LDL-C = low-density lipoprotein cholesterol; N/A = not available; SBP = systolic blood pressure; SD = standard deviation; TC = total cholesterol; TG = triglycerides; TRE = time-restricted feeding; VLCD = very low-calorie diet; WC = waist circumference.*

*RCT = randomized controlled trial*

**Figure 3.**
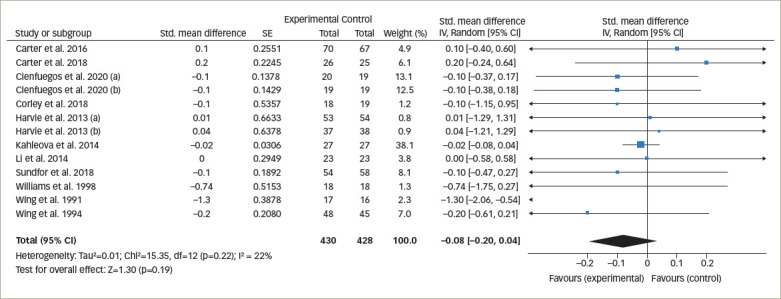
Forest plot comparing the effects of intermittent fasting versus control on glycated haemoglobin levels

*CI = confidence interval; df = degrees of freedom; IV= inverse variance; SE = standard error; Std = standardized. Cienfuegos et al. 2020 (a): 4 hours time restricted feeding intervention; Cienfuegos et al. 2020 (b): 6 hours time restricted feeding intervention; Harvie et al. 2013 (a): Data at 3 months; Harvie et al. 2013 (b): Data at 4 months.*

**Figure 4.**
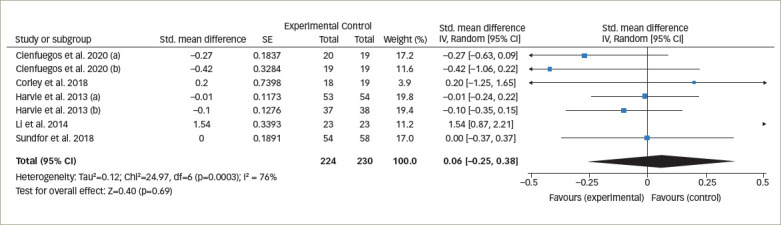
Forest plot comparing the effects of intermittent fasting versus control on fasting blood sugar

*CI = confidence interval; df = degrees of freedom; IV= inverse variance; SE = standard error; Std = standardized. Cienfuegos et al. 2020 (a): 4 hours time restricted feeding intervention; Cienfuegos et al. 2020 (b): 6 hours time restricted feeding intervention; Harvie et al. 2013 (a): Data at 3 months; Harvie et al. 2013 (b): Data at 4 months.*

### Outcome measures

#### Glycaemic control (glycated haemoglobin and fasting blood glucose levels)

*Figure 3* represents the overall analysis of 11 studies (13 arms) to measure the effect of IF on patients’ HbA1c level. There was no statistically significant difference between IF and control groups (statistically meaningful difference [SMD] -0.08, 95% CI -0.20 to 0.04;p=0.19, I^2^=22%). Overall, seven studies on patients’ fasting blood glucose value (FBG) were analysed, and the meta-analysis revealed no significant difference between the IF and control groups (SMD 0.06, 95% CI -0.25 to 0.38;p=0.69, I^2^=76%; *Figure 4*).

### Sensitivity analysis

#### Glycated haemoglobin level

**Figure 5.**
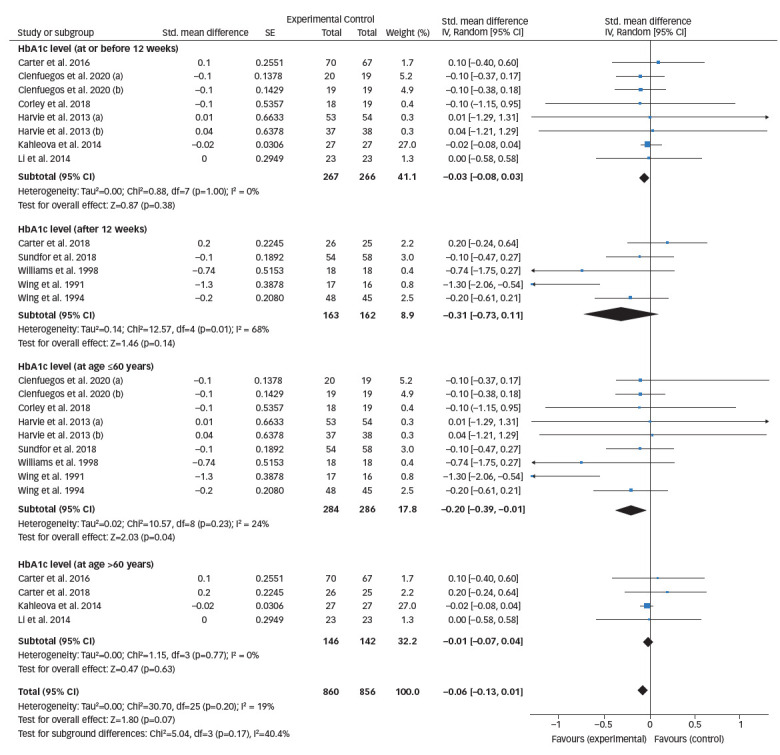
Forest plot comparing glycated haemoglobin outcomes based on treatment duration (before and after 12 weeks of treatment) and age (≤60 years and >60 years)

Five studies monitored HbA1c levels after 12 weeks’ treatment duration, with the remaining eight studies monitoring HbA1c level at ≤12 weeks’ treatment duration. When analysed by treatment duration, there was still no significant difference between the IF and control groups (SMD -0.03, 95% CI -0.08 to 0.03;p=0.38, I^2^=0%). Further, meta-analysis was performed including only the five studies with reported HbA1c level after 12 weeks’ treatment (SMD -0.31, 95% CI -0.73 to 0.11;p=0.14, I^2^=68%; *Figure 5*).

Four studies included participants >60 years of age and nine studies included those ≤60 years of age. When analysed by patient age, the reported change in the HbA1c levels of IF and control groups was statistically significant (SMD -0.20, 95% CI -0.39 to -0.01;p=0.04, I^2^=24%; *Figure 5*).

#### Fasting blood sugar levels

**Figure 6.**
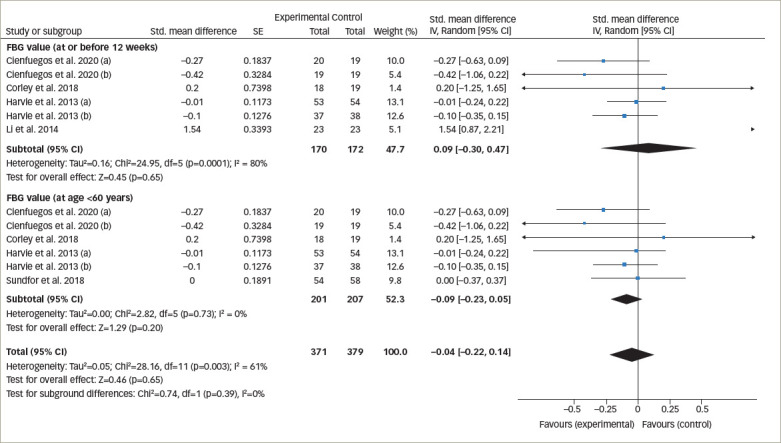
Forest plot comparing fasting blood glucose outcomes based on treatment duration (≤12 weeks of treatment) and age (≤60 years)

One study monitored FBG after 6 months, and six studies monitored FBG at ≤6 months. When the >6 months study was excluded from analysis, there was still no significant difference between the FBG values of the IF and control groups (SMD 0.09, 95% CI -0.30 to 0.47;p=0.65, I^2^ =80%; *Figure 6*).

Similarly, one study had participants over the age of 60 years, and the remaining six had participants aged ≤60 years. When the >60 years of age study was excluded, there was no significant difference in the FBG values of the IF and control groups (SMD -0.09, 95% CI -0.23 to 0.05;p=0.20, I^2^=0%; *Figure 6*).

*CI = confidence interval; df = degrees of freedom; IV= inverse variance; SE = standard error; Std = standardized. Cienfuegos et al. 2020 (a): 4 hours time restricted feeding intervention; Cienfuegos et al. 2020 (b): 6 hours time restricted feeding intervention; Harvie et al. 2013 (a): Data at 3 months; Harvie et al. 2013 (b): Data at 4 months.*

### Publication bias

**Figure 7.**
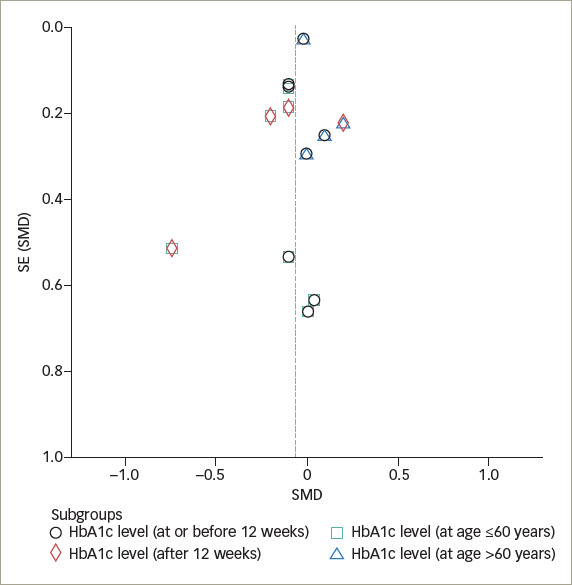
Funnel plot of glycated haemoglobin level

The funnel plot illustrating the effect of IF on glycaemic control demonstrates a pattern that is nearly symmetric (*Figure 7* *and* *8*), which suggests that the findings were less likely to be influenced by publication bias.

## Discussion

IF has gained recognition as a method to improve metabolic health. In IF, eating habits are based on eating very few or no calories during periods from 12 hours to many days, while following a regular routine. An imbalance in the levels of adiponectin and leptin is a factor in the altered metabolism that increases the risk of developing T2DM.^37,38^ Interestingly, various studies have shown that IF leads to lower leptin levels, as well as higher adiponectin levels, which can improve insulin resistance.^37–39^

We have gathered clinical trials on the impact of IF on glycaemic control among patients with T2DM. There were only a handful of studies that focused on the effect of IF on metabolism of lipids which was not included in our meta-analysis. Also, these studies varied in terms of participants’ age, duration of IF, restriction of calorie intake and timing of outcome measurements. Hence, this meta-analysis was designed to test whether IF significantly impacted patients’ HbA1c and FBG levels, with a pooled analysis of outcomes measured at different intervals. Overall, there was no significant change in patients’ HbA1c and FBG levels between IF and control.

In one study, significant reductions in HbA1c and weight were reported for almost all patients.^19^ Also, there were no side effects noted among the patients on IF. Similarly, another meta-analysis also reported the positive impact of IF on reducing HbA1c level and body weight.^40,41^ These results are in line with some of the studies included in this review, which have reported that IF is superior to control group in terms of weight reduction and glycaemic control.^30–32,35,36^ On the contrary, other included studies showed no significant difference in HbA1c levels following IF versus the control group.^27–29,33,34^ Another meta-analysis has also concluded that there was no significant impact of IF on patients’ HbA1c levels, although IF may be useful in preventing metabolic disorders.^40^ These results are in line with the present meta-analysis, which reports no significant difference in glycaemic control between patients using IF versus another intervention.^42,43^ Our meta-analysis on performing sensitivity analysis found that benefits of IF also depend on participants’ age as IF was well tolerated among patients aged ≤60 years and has a significant impact on HbA1c levels in this age group. There was no significant impact of IF on HbA1c among patients over the age of 60 years.

*CI = confidence interval; df = degrees of freedom; HbA1c = glycated haemoglobin; IV= inverse variance; SE = standard error; Std = standardized. Cienfuegos et al. 2020 (a): 4 hours time restricted feeding intervention; Cienfuegos et al. 2020 (b): 6 hours time restricted feeding intervention; Harvie et al. 2013 (a): Data at 3 months; Harvie et al. 2013 (b): Data at 4 months*

*HbA1c = glycated haemoglobin; SE = standard error; SMD = standardized mean difference.*

**Figure 8.**
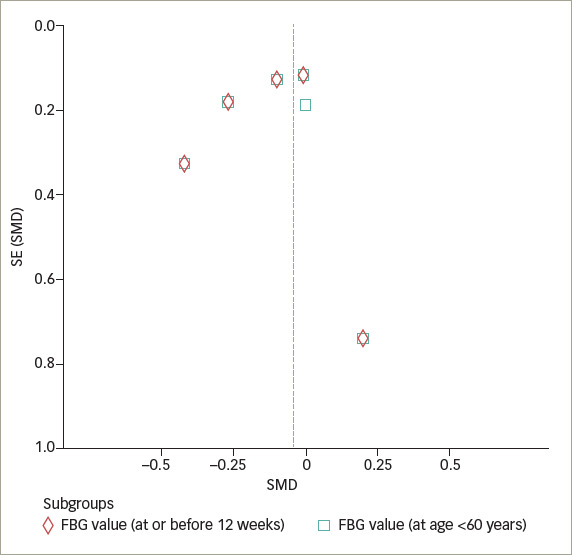
Funnel plot of fasting blood sugar level

*FBG = fasting blood glucose; SE = standard error; SMD = standardized mean difference*

Pooled analysis of the studies included in our meta-analysis showed no significant reduction in patients’ FBG levels following IF. On the contrary, a study where IF was followed for 12 months reported significant reductions in fasting insulin levels and homeostatic model assessment of insulin resistance (HOMA-IR) levels in the alternate-day fasting group.^44^ Similarly, another systematic review found that IF reduces participants’ FBG levels.^41^ Studies have also reported that insulin decreases because of increased insulin sensitivity and, hence, decrease fasting and postprandial blood glucose in patients with diabetes.^40,44^

A few studies comment that IF and continuous energy restrictions have equal benefits in achieving long-term weight and glycaemic control.^19,40,42,44^ The IF diet differs from the ketogenic or low-calorie diet in that it does not restrict carbohydrate intake; therefore, the direct effect on blood glucose levels in the short-term is unknown. Yet, IF is certainly beneficial in regulating blood glucose levels during fasting. IF can enhance insulin sensitivity in the long-term and therefore needs to be practised by patients with diabetes. Moreover, it is important to follow standard guidelines while practicing any dietary restrictions to avoid serious adverse effects.

There were some limitations on this analysis. First, there is heterogeneity among the studies and dietary interventions, with the treatment duration likely being the primary source of heterogeneity. As a result, the randomeffects models were used in this analysis for merging, and sensitivity analyses were carried out in accordance with potential sources. Second, there were only a small number of randomized controlled trials that met the inclusion criteria, and sample sizes were small. It was also difficult to reach a firm conclusion about how IF affected glycaemic control, because the intervention duration ranged from 8 weeks to 12 months. Additionally, it was not possible to determine whether IF was safe for patients with T2DM who were taking insulin in our analyses, which is particularly important.

## Conclusion

IF and usual diet pattern have no difference in terms of glycaemic control. Although, IF might be used as a preventative diet pattern in the pre-diabetic population, as it works well in the long-term to achieve controlled sugar levels. It is clearly evident from our meta-analysis that IF alone does not reduce blood glucose levels in patients with diabetes. Further clinical trials are required with uniform or standard IF intervention to study its impact in depth.

## References

[1] Saeedi P, Petersohn I, Salpea P (2019). Global and regional diabetes prevalence estimates for 2019 and projections for 2030 and 2045: Results from the International Diabetes Federation Diabetes Atlas, 9th edition.. Diabetes Res Clin Pract..

[2] Willcox M, Elugbaju C, Al-Anbaki M (2021). Effectiveness of medicinal plants for glycaemic control in type 2 diabetes: An overview of meta-analyses of clinical trials.. Front Pharmacol..

[3] Ramtahal R, Khan C, Maharaj-Khan K (2015). Prevalence of self-reported sleep duration and sleep habits in type 2 diabetes patients in South Trinidad.. J Epidemiol Glob Health..

[4] Franz MJ, Boucher JL, Rutten-Ramos S, VanWormer JJ (2015). Lifestyle weight-loss intervention outcomes in overweight and obese adults with type 2 diabetes: A systematic review and meta-analysis of randomized clinical trials.. J Acad Nutr Diet..

[5] World Health Organization. Diabetes.. http://www.who.int/news-room/fact-sheets/detail/diabetes.

[6] Tinsley GM, La Bounty PM (2015). Effects of intermittent fasting on body composition and clinical health markers in humans.. Nutr Rev..

[7] Varady KA, Bhutani S, Church EC, Klempel MC (2009). Short-term modified alternate-day fasting: A novel dietary strategy for weight loss and cardioprotection in obese adults.. Am J Clin Nutr..

[8] Bhutani S, Klempel MC, Berger RA, Varady KA (2010). Improvements in coronary heart disease risk indicators by alternate-day fasting involve adipose tissue modulations.. Obesity..

[9] Klempel MC, Kroeger CM, Varady KA (2013). Alternate day fasting increases LDL particle size independently of dietary fat content in obese humans.. Eur J Clin Nutr..

[10] Tinsley GM, Forsse JS, Butler NK (2017). Time-restricted feeding in young men performing resistance training: A randomized controlled trial.. Eur J Sport Sci..

[11] Cioffi I, Evangelista A, Ponzo V (2018). Intermittent versus continuous energy restriction on weight loss and cardiometabolic outcomes: A systematic review and meta-analysis of randomized controlled trials.. J Transl Med..

[12] Patterson RE, Sears DD (2017). Metabolic effects of intermittent fasting.. Annu Rev Nutr..

[13] Allaf M, Elghazaly H, Mohamed OG (2019). Intermittent fasting for the prevention of cardiovascular disease.. Cochrane Database Syst Rev..

[14] Adler-Lazarovits C, Weintraub A (2019). Physicians’ attitudes and views regarding religious fasting during pregnancy and review of the literature.. Europ J Obst Gynecol Reproduct Biol..

[15] Lowe DA, Wu N, Rohdin-Bibby L (2020). Effects of time-restricted eating on weight loss and other metabolic parameters in women and men with overweight and obesity: The TREAT randomized clinical trial.. JAMA Intern Med..

[16] Sutton EF, Beyl R, Early KS (2018). Early time-restricted feeding improves insulin sensitivity, blood pressure, and oxidative stress even without weight loss in men with prediabetes.. Cell Metab..

[17] Harvie MN, Pegington M, Mattson MP (2011). The effects of intermittent or continuous energy restriction on weight loss and metabolic disease risk markers: A randomized trial in young overweight women.. Int J Obes..

[18] Antoni R, Johnston KL, Collins AL, Robertson MD (2018). Intermittent v. continuous energy restriction: Differential effects on postprandial glucose and lipid metabolism following matched weight loss in overweight/obese participants.. Br J Nutr..

[19] Furmli S, Elmasry R, Ramos M, Fung J (2018). Therapeutic use of intermittent fasting for people with type 2 diabetes as an alternative to insulin.. BMJ Case Rep..

[20] Ash S, Reeves MM, Yeo S (2003). Effect of intensive dietetic interventions on weight and glycaemic control in overweight men with type II diabetes: A randomised trial.. Int J Obes Relat Metab Disord..

[21] Parvaresh A, Razavi R, Abbasi B (2019). Modified alternate-day fasting vs. calorie restriction in the treatment of patients with metabolic syndrome: A randomized clinical trial.. Complement Ther Med..

[22] Wing RR, Blair E, Marcus M (1994). Year-long weight loss treatment for obese patients with type II diabetes: Does including an intermittent very-low calorie diet improve outcome?. Am J Med..

[23] Carlson O, Martin B, Stote KS (2007). Impact of reduced meal frequency without caloric restriction on glucose regulation in healthy, normal-weight middle-aged men and women.. Metabolism..

[24] Soeters MR, Lammers NM, Dubbelhuis PF (2009). Intermittent fasting does not affect wholebody glucose, lipid, or protein metabolism.. Am J Clin Nutr..

[25] McInnes MDF, Moher D, Thombs BD (2018). Preferred reporting items for a systematic review and meta-analysis of diagnostic test accuracy studies: The PRISMA-DTA statement.. JAMA..

[26] Higgins JPT, Thomas J, Chandler J (2019). Cochrane Handbook for Systematic Reviews of Interventions..

[27] Cienfuegos S, Gabel K, Kalam F (2020). Effects of 4-and 6-h time-restricted feeding on weight and cardiometabolic health: A randomized controlled trial in adults with obesity.. Cell Metab..

[28] Carter S, Clifton PM, Keogh JB (2018). Effect of intermittent compared with continuous energy restricted diet on glycemic control in patients with type 2 diabetes: A randomized noninferiority trial.. JAMA Netw Open..

[29] Carter S, Clifton PM, Keogh JB (2016). The effects of intermittent compared to continuous energy restriction on glycaemic control in type 2 diabetes; a pragmatic pilot trial.. Diabetes Res Clin Pract..

[30] Corley B, Carroll R, Hall R (2018). Intermittent fasting in type 2 diabetes mellitus and the risk of hypoglycaemia: A randomized controlled trial.. Diabet Med..

[31] Harvie M, Wright C, Pegington M (2013). The effect of intermittent energy and carbohydrate restriction v. daily energy restriction on weight loss and metabolic disease risk markers in overweight women.. Br J Nutr..

[32] Kahleova H, Belinova L, Malinska H (2014). Eating two larger meals a day (breakfast and lunch) is more effective than six smaller meals in a reduced-energy regimen for patients with type 2 diabetes: A randomised crossover study.. Diabetologia..

[33] Li C, Sadraie B, Steckhan N (2017). Effects of a one-week fasting therapy in patients with type-2 diabetes mellitus and metabolic syndrome – a randomized controlled explorative study.. Exp Clin Endocrinol Diabetes..

[34] Sundfør T, Svendsen M, Tonstad S (2018). Effect of intermittent versus continuous energy restriction on weight loss, maintenance and cardiometabolic risk: A randomized 1-year trial.. Nutr Metab Cardiovasc..

[35] Williams KV, Mullen ML, Kelley DE, Wing RR (1998). The effect of short periods of caloric restriction on weight loss and glycemic control in type 2 diabetes.. Diabetes Care..

[36] Wing RR, Marcus MD, Salata R (1991). Effects of a very-low-calorie diet on long-term glycemic control in obese type 2 diabetic subjects.. Arch Intern Med..

[37] Anton SD, Moehl K, Donahoo WT (2018). Flipping the metabolic switch: Understanding and applying the health benefits of fasting.. Obesity..

[38] Zubrzycki A, Cierpka-Kmiec K, Kmiec Z, Wronska A (2018). The role of low-calorie diets and intermittent fasting in the treatment of obesity and type-2 diabetes.. J Physiol Pharmacol..

[39] Cho Y, Hong N, Kim KW (2019). The effectiveness of intermittent fasting to reduce body mass index and glucose metabolism: A systematic review and meta-analysis.. J Clin Med..

[40] Yuan X, Wang J, Yang S (2022). Effect of intermittent fasting diet on glucose and lipid metabolism and insulin resistance in patients with impaired glucose and lipid metabolism: A systematic review and meta-analysis.. Int J Endocrinol..

[41] Yang F, Liu C, Liu X (2021). Effect of epidemic intermittent fasting on cardiometabolic risk factors: A systematic review and meta-analysis of randomized controlled trials.. Front Nutr..

[42] Wang X, Li Q, Liu Y (2021). Intermittent fasting versus continuous energy-restricted diet for patients with type 2 diabetes mellitus and metabolic syndrome for glycemic control: A systematic review and meta-analysis of randomized controlled trials.. DiabetesRes Clin Pract..

[43] Welton S, Minty R, O’Driscoll T (2020). Intermittent fasting and weight loss: Systematic review.. Can Fam Physician..

[44] Gabel K, Kroeger CM, Trepanowski JF (2019). Differential effects of alternate-day fasting versus daily calorie restriction on insulin resistance.. Obesity..

